# Mapping the Overlap of Poverty Level and Prevalence of Diagnosed Chronic Kidney Disease Among Medicare Beneficiaries in the United States

**DOI:** 10.5888/pcd21.230286

**Published:** 2024-04-11

**Authors:** Yun Han, Fang Xu, Hal Morgenstern, Jennifer Bragg-Gresham, Brenda W. Gillespie, Diane Steffick, William H. Herman, Meda E. Pavkov, Tiffany Veinot, Rajiv Saran

**Affiliations:** 1 Department of Internal Medicine, Division of Nephrology, University of Michigan, Ann Arbor; 2 Division of Diabetes Translation, National Center for Chronic Disease Prevention and Health Promotion, Centers for Disease Control and Prevention, Atlanta, Georgia; 3 Department of Epidemiology, University of Michigan, Ann Arbor; 4 Departments of Environmental Health Sciences and Urology, University of Michigan, Ann Arbor; 5 Department of Biostatistics, University of Michigan, Ann Arbor; 6 Department of Internal Medicine, Division of Metabolism, Endocrinology, and Diabetes, University of Michigan, Ann Arbor; 7 School of Information, University of Michigan, Ann Arbor

**Figure:**
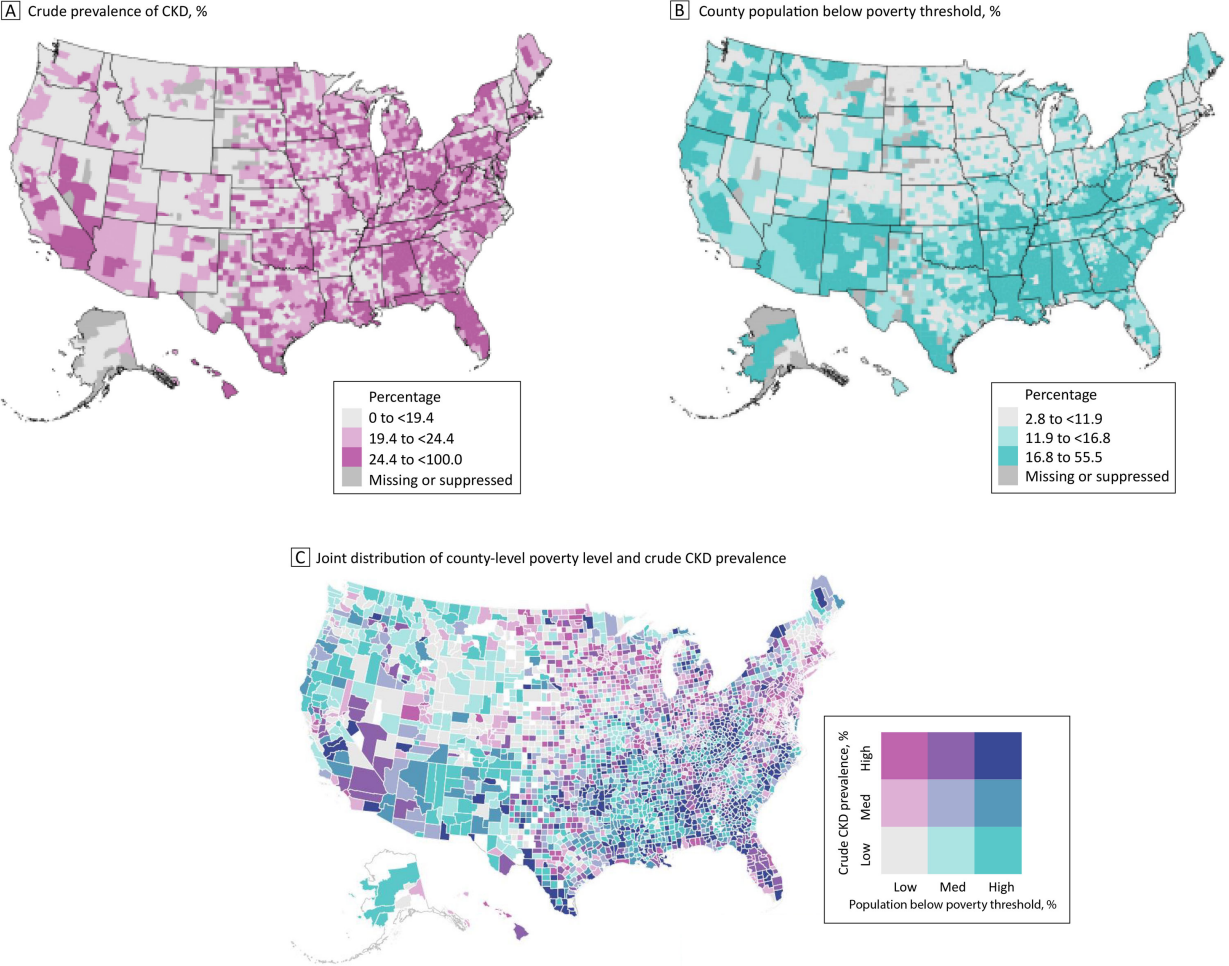
Geographic differences by county in CKD prevalence among US Medicare beneficiaries aged ≥65 years and in poverty levels, with higher rates of CKD in Florida and Appalachia (Panel A) and higher poverty levels in the Southeast (Panel B). Many counties in the South have both high poverty levels and a high prevalence of CKD, while many counties in the Northeast and Midwest have lower poverty levels but a high prevalence of CKD (Panel C). Abbreviation: CKD, chronic kidney disease. Data sources: Centers for Disease Control and Prevention (9); US Census Bureau (11).

## Background

Living in high-poverty neighborhoods has been identified as a contributing factor to the development and progression of chronic kidney disease (CKD) (1,2). High-poverty neighborhoods often face inequities related to social determinants of health, such as lower incomes; gaps in educational achievement; inadequate access to healthy food, health care, green space, and high-quality recreational facilities; and greater exposure to air and water pollution (3–8). A limited ability to purchase healthy food and access recreational facilities and preventive health care can delay diagnosis and timely management of CKD. Understanding the distribution of CKD at the county level in relation to poverty level is important: this knowledge can guide population-level interventions for CKD prevention and management. The objectives of this study were to examine the county-level association between poverty level and diagnosed CKD and to illustrate county-level bivariate distribution of poverty and CKD among Medicare fee-for-service beneficiaries aged 65 years or older in the US.

## Methods

We calculated the county-level prevalence of diagnosed CKD in each US county among Medicare fee-for-service beneficiaries aged 65 years or older based on 5% claims data for 2019. The study population included beneficiaries who had full-year Parts A and B enrollment and at least 1 inpatient or outpatient visit in 2019. The numerator of CKD prevalence included eligible beneficiaries with at least 1 claim in 2019 containing an ICD-10-CM (*International Classification of Diseases, Tenth Revision, Clinical Modification*) diagnosis code for CKD (9,10). We excluded beneficiaries with end-stage kidney disease because they are not at risk for CKD. We also excluded Medicare beneficiaries covered by Part C (managed care/Medicare Advantage plans) because of limited availability of data. The total study population consisted of 1,234,056 beneficiaries in 3,097 counties (98.5% of all 3,143 US counties).

Poverty level was measured as the percentage of the total county population below the poverty threshold extracted from the American Community Survey 5-year data (2015–2019) (11).

We linked measurements of CKD and poverty by using county Federal Information Processing Standards (FIPS) codes. We standardized county-level prevalence of CKD based on strata of demographic characteristics. The 5% sample of the 2019 Medicare population aged 65 years or older (ie, the study population) served as the standard population. We performed 3 analyses: 1) a crude (unstandardized) analysis; 2) standardization on age alone (age categories 65–69, 70–79, 80–89, ≥90 y); and 3) standardization on age, sex (male, female), and race and ethnicity (White, Black, Hispanic, Asian, other [American Indian or Alaska Native, Native Hawaiian or Other Pacific Islander, other], unknown).

We then generated 2 univariate choropleth (color-coded or shaded) maps to separately depict crude county-level distributions of CKD prevalence and poverty levels across the US. In addition, we created a bivariate map (in R version 4.3.1 [R Foundation for Statistical Computing]) that combines the distributions of both variables in each county by using a 2-dimensional (3 × 3) key to show the tertile (high, medium, low) of CKD prevalence and poverty level. Thus, the color of each county represents the association between county poverty level and CKD prevalence, and the bivariate map shows the pattern of those associations across the US, emphasizing the clustering and geographic patterns of counties. Data were suppressed for counties with 10 or fewer beneficiaries (n = 108, 3.4% of all counties) according to the Centers for Medicare & Medicaid Services small-cell suppression rule to protect privacy (12). This suppression had only a minor effect on the map’s appearance, but it may have led to underrepresentation of counties with smaller populations of older adults.

## Highlights

The mean (SD) county-level crude prevalence of CKD in the study population was 22.1% (6.5%). The mean (SD) prevalence of poverty was 15.4% (6.9%). As the poverty level increased, the crude prevalence of CKD also rose, from 20.9% to 23.4% (Table). This pattern was nearly the same when standardized measures were used (Table), suggesting that age, sex, and race and ethnicity did not confound the association between poverty-level tertile and CKD prevalence.

**Table:** Crude and Standardized Prevalence of Diagnosed Chronic Kidney Disease Among Medicare Beneficiaries Aged ≥65 Years, by Tertile of County Poverty Level (N = 3,097), United States, 2019^a^

Prevalence	All	Tertile of county poverty level^b^		
		Low	Medium	High
Crude	22.1 (6.5)	20.9 (6.0)	22.1 (6.2)	23.4 (7.0)
Standardized for age^c^	22.2 (6.6)	21.0 (6.2)	22.2 (6.3)	23.5 (7.1)
Standardized for age, sex, and race and ethnicity^c^	22.0 (6.9)	20.8 (6.4)	22.0 (6.4)	23.0 (7.6)

Abbreviation: CKD, chronic kidney disease.

a Data source: 2019 Medicare 5% Sample Data and American Community Survey data (2015−2019) (11). All values are mean (SD).

b Tertile breaks were used to create the categories for all data from the study population for poverty level (expressed as percentage of the population below the federal poverty threshold): low, < 12%; medium, 12%–17%; high, > 17%).

c Standardization was based on strata for age (65−70, 70−80, 80−90, >90 y), sex, and race and ethnicity (White, Black, Hispanic, Asian, other [American Indian/Alaska Native, Native Hawaiian/Other Pacific Islander, other], unknown). The standard population was Medicare fee-for-service beneficiaries aged ≥65 years in 2019.

We observed considerable geographic variations in crude CKD prevalence (Figure A) and poverty level (Figure B). CKD prevalence was higher in Florida and the Appalachian region, which encompasses parts of Ohio, Pennsylvania, West Virginia, Kentucky, Tennessee, and Alabama (Figure A). Poverty levels were clustered, with a high concentration of counties east of the Mississippi River having higher poverty levels (Figure B).

The bivariate map (Figure C) shows the underlying joint distribution of county-level poverty and CKD prevalence. Many counties in the Southeast show high levels of both poverty and CKD, and many counties in New England show low levels of both poverty and CKD. Both patterns indicate a positive association between the 2 measures (high–high or low–low). In contrast, several counties in the mid-Atlantic coast and the upper Midwest show high CKD prevalence and low poverty level, and many counties in the West show high levels of poverty level and low CKD prevalence, indicating an inverse association between the 2 measures (high–low or low–high).

## Action

The observed spatial disparities in CKD and poverty suggest that a one-size-fits-all intervention may not be effective in decreasing the prevalence of CKD. Tailored interventions for older adults are necessary. In high-prevalence/high-poverty counties, interventions could focus on local challenges among older adults by improving health care access, addressing socioeconomic barriers to health, and implementing strategies such as subsidized healthy food programs and enhanced health care services. Conversely, in high-prevalence/low-poverty counties, strategies could encompass health education and disease management programs, with a focus on public awareness campaigns about CKD risk factors and promotion of regular health screenings. In these counties, factors other than economic status, including prevalence of comorbidities, health care access, environmental conditions, and lifestyle choices, may influence the prevalence of CKD.

Although our study sheds light on the correlation between county-level poverty and the prevalence of CKD, it has limitations in addressing the multifaceted nature of poverty. First, individual-level poverty, which encompasses personal financial constraints and limited access to health care, also plays a crucial role in CKD risk. Our focus on county-level data may not fully capture individual poverty experiences and their direct effect on CKD. Studies incorporating individual-level socioeconomic data could enhance the understanding of the complex interplay between poverty and CKD prevalence. Second, we identified CKD based on ICD-10-CM diagnosis codes because we lacked laboratory data (eg, estimated glomerular filtration rate). This reliance on diagnosis codes may have resulted in an underestimation of actual CKD prevalence and possible distortions in observed geographic patterns. A third limitation is the choice of geographic unit; using county-level data may mask finer-scale variations and socioeconomic disparities, particularly in urban areas. Fourth, our cross-sectional study examined the relationship between county-level poverty and CKD prevalence at a single time point. As highlighted by Lapedis et al (13), a cross-sectional approach may not fully encapsulate the complex and evolving relationship between neighborhood characteristics and the various stages of CKD, particularly the early stages. The reliance on a single time-point analysis limits our ability to understand these dynamics over the life course. Overall, further research, accounting for confounders and mediators, may be essential to delve into the underlying causes of the observed spatial disparities in CKD and poverty. This research includes identifying factors contributing to high CKD prevalence in low-poverty counties in the Northeast and Midwest. These findings may guide clinical practice and health policy aimed at mitigating CKD disparities across the US.
